# Poly herbal formulation with anti-elastase and anti-oxidant properties for skin anti-aging

**DOI:** 10.1186/s12906-018-2097-9

**Published:** 2018-01-29

**Authors:** Induja Kalyana Sundaram, Deepika Deeptirekha Sarangi, Vignesh Sundararajan, Shinomol George, Sahabudeen Sheik Mohideen

**Affiliations:** 1 Department of Biotechnology, Dayanandasagar College of Engineering, Kumaraswamy Layout, Bangalore, 560078 Karnataka India; 2 Department of Biotechnology, School of Bioengineering, SRM Institute of Science and Technology, Kattankulathur, Chennai, Tamil Nadu 603203 India

**Keywords:** Oxidative stress, Reactive oxygen species, Skin anti-aging, Anti-oxidant activity, Anti-elastase activity, Poly herbal formulations, Nitric oxide scavenging assay, Anti-elastase assay

## Abstract

**Background:**

Skin forms an important part of human innate immune system. Wrinkles, thinning and roughening of skin are some of the symptoms that affect the skin as it ages. Reactive oxygen species induced oxidative stress plays a major role in skin aging by modulating the elastase enzyme level in the skin. Extrinsic factors that affect skin aging such as UV radiation can also cause malignant melanoma. Here we selected four medicinal plant materials, namely, leaves of *Nyctanthes arbor-tristis,* unripe and ripe *Aegle marmelos* fruit pulp and the terminal meristem of *Musa paradisiaca* flower and investigated their anti-aging properties and cytotoxicity in vitro individually as well as in a poly herbal formulation containing the four plant extracts in different ratios.

**Methods:**

The phytochemical contents of the plant extracts were investigated for radical scavenging activity and total reducing power. Based upon its anti-oxidant properties, a poly herbal formulation containing leaves of *Nyctanthes arbor-tristis*, unripe and ripe fruit pulp of *Aegle marmelos*, and the terminal meristem of *Musa paradisiaca* flower in the ratio 6:2:1:1 (Poly Herbal Formulation 1) and 1:1:1:1 (Poly Herbal Formulation 2), respectively were formulated.

**Result:**

It has been observed that the Poly Herbal Formulation 1 was more potent than Poly Herbal Formulation 2 due to better anti-oxidant and anti-elastase activities in NIH3T3 fibroblast cells. In addition Poly Herbal formulation 1 also had better anti-cancer activity in human malignant melanoma cells.

**Conclusion:**

Based on these results these beneficial plant extracts were identified for its potential application as an anti-aging agent in skin creams as well as an anti-proliferation compound against cancer cells.

**Electronic supplementary material:**

The online version of this article (10.1186/s12906-018-2097-9) contains supplementary material, which is available to authorized users.

## Background

Skin aging is mainly characterized by wrinkle formation, uneven pigmentation, darkening, thinning, sagging, and roughening of skin [1, 2].This could be caused either by intrinsic or extrinsic mechanisms [3–5]. Intrinsic skin aging is inevitable and occurs as age progresses [6, 7]. It mainly occurs due to cumulative effects of oxidative stress. Reactive oxygen species (ROS) (free radicals) are produced during normal cellular metabolism and are needed for normal biological functions. However, higher amounts of ROS such as hydrogen peroxide, superoxide, and peroxynitrite radicals cause oxidative stress, which damages DNA, RNA, lipids and proteins in skin, and cause skin cancer [8]. Extrinsic skin aging mainly occurs due to environmental factors such as UV irradiation, physical stress, nutritional deficiency, and alcohol consumption and thus can be controlled [9–12]. UV irradiation increases ROS production, accelerates aging process and telomere shortening [3, 13]. As both intrinsic and extrinsic mechanisms of skin aging are caused due to oxidative stress, the use of anti-oxidants can be an effective approach to treat skin aging and skin aging-related problems [14].

Malignant melanoma is a type of skin cancer that develops in melanocytes that are specialized cells that impart color to the skin via melanin secretion [15]. Globally, 132,000 patients are diagnosed with melanoma each year [16]. Current treatment of malignant melanoma includes surgery, radiation therapy, immunotherapy, chemotherapy, and targeted therapy [17]. Phytochemicals are important constituents of human food sources and therefore, investigation of the anti-oxidant, anti-elastase and anti-proliferative properties of polyphenolic compounds present in commonly consumed fruits such as *Aegle marmelos (common name – bael)* and *Musa paradisiaca (common name – banana)* would be a more effective approach to treat age-related diseases and skin cancer [18, 19].

Studies have reported that *Aegle marmelos* exhibits anti-viral effect [20], inhibitory effect against Ehrlich ascites carcinoma [21], anti-hyperglycemic effect [22, 23], anti-adipogenic effect [24], anti-diarrhoeal effect [25], chemo-modulatory effect against DMBA-induced skin tumorigenesis [26], ameliorative effect against alloxan-induced diabetic cardiomyopathy [27], radioprotective effect [28], anti-oxidant and hepatoprotective effects [29]. In addition, consumption of unripe and half ripe *Aegle marmelos* helps in complete digestion of food, prevents scurvy and has anti-microbial activity against pathogenic intestinal microorganism [30]. *Nyctanthes arbor-tristis* (common name – night flowering jasmine) exhibits anti-inflammatory effect [31], anti-amoebic activity, anti-leishmanial activity, anti-viral activity [32], analgesic activity, anti-pyretic activity and ulcerogenic activity [33], anti-oxidant activity [34, 35], hypoglycemic activity, and hypolipidemic activity [36] in vitro studies. *Musa paradisiaca* is one of the world’s leading food crop [37] and exhibits leishmanicidal activity [38], anti-oxidant activity [39, 40], hepatoprotective activity [41], anti-diarrhoeal activity [42], anti-ulcerogenic activity [40], anti-hyperglycemic effect [43], and anti-coccidial activity [44]. However, studies have not been performed to determine the anti-aging and anti-carcinogenic effects of *Aegle marmelos*, *Nyctanthes arbor-tristis* and *Musa paradisiaca*. Moreover, there is no study till date that has investigated the aforementioned properties of the plant extracts when added as a formulation.

Thus, the current study was performed to investigate the anti-oxidative capacities of unripe fruit pulp of *Aegle marmelos*, ripe fruit pulp of *Aegle marmelos*, leaves of *Nyctanthes arbor-tristis,* and the terminal meristem of *Musa paradisiaca* flower. Based on the anti-oxidative capacities of plant extracts, poly herbal formulations were prepared and tested for their anti-oxidant activity and anti-elastase inhibition capacity using biochemical assays, and cytotoxicity was investigated using normal fibroblast NIH3T3 cell line and human malignant melanoma A375 cell line. It was found that PHF1 was more potent than PHF2 in its anti-oxidant, anti-elastase and cytotoxic properties.

## Methods

### Chemicals and reagents

2,2-Diphenyl-1-picrylhydrazyl (DPPH), methanol, ascorbic acid, sodium nitroprusside, curcumin, sodium chloride, Griess reagent, porcine pancreatic elastase (EC.3.4.21.36), N-succinyl-Ala-Ala-Ala-p-nitroanilide, tris base, hydrochloric acid, copper sulphate, ferric chloride, trichloroacetic acid, potassium ferricyanide, dimethyl sulfoxide (DMSO), 3-(4,5-Dimethyl-2-thiazolyl)-2,5-diphenyl-2H-tetrazolium bromide (MTT), Dulbecco’s Modified Eagle Medium (DMEM), fetal bovine serum (FBS), Mayer’s reagent, lead acetate, chloroform, acetic acid, and sulphuric acid were purchased from Sigma-Aldrich (Bangalore, India). NIH3T3 mouse embryonic fibroblast cell line and A375 human malignant melanoma cancer cell lines were purchased from American Type Culture Collection (Rockville, MD, USA).

### Plant material collection and extract preparation

The anti-oxidant capacity, anti-aging capacity and cytotoxicity of unripe and ripe fruit pulp of *Aegle marmelos*, leaves of *Nyctanthes arbor-tristis*, terminal meristem of *Musa paradisiaca* flower, and their poly herbal formulations were investigated in this study. Unripe and ripe fruits of *Aegle marmelos* were collected from Minerva circle temple garden, Bengaluru, Karnataka, India. *Musa paradisiaca* flowers were collected from Harohalli farmlands, Bengaluru, Karnataka, India. *Nyctanthes arbor-tristis* leaves were collected locally from Bengaluru, Karnataka, India. All the collected plant materials were deposited and authenticated by Regional Ayurveda Research Institute for Metabolic Disorders, Ministry of AYUSH, Government of India, Bengaluru, India. An authentication certificate is attached in the final page of Additional file 1.

The plant materials were cleaned, shade dried for eight days, finely powdered and used for extraction, as described previously [45]. Pure methanol was used to prepare 20 (*w*/*v*) % plant extract solutions. Briefly, 50 mL of each of the plant extracts were transferred into a 500 mL beaker and sealed with aluminum foil and kept in a water bath for 4 h at 50 °C. The obtained liquid extracts were filtered using Whatman filter paper (11 μm). Then the filtrates were transferred into a 50 mL beaker and sealed with aluminum foil and kept in a water bath at 80 °C until semi solid phase extracts were obtained. The extracts were stored in aliquots in dark for further use.

### Preliminary qualitative phytochemical analysis of plant extracts

In order to determine the presence of various phytochemicals in the plant extracts, 12% (*w*/*v*) aqueous plant extracts were prepared from methanolic plant extracts [46–49].

### Test for alkaloids (Mayer’s test)

To 3 mL of 1% hydrochloric acid, 3 mL of aqueous plant extract was added and kept in a water bath at 50 °C for few minutes. To this solution, 1 mL of Mayer’s reagent was added. The presence of turbidity in the resulting precipitate indicated the presence of alkaloids.

### Test for flavonoids

To 1 mL of 10% lead acetate solution, 1 mL of aqueous plant extract was added. The formation of a yellow precipitate indicated the presence of flavonoids.

### Test for glycosides (Liebermann’s test)

To 2 mL of chloroform and acetic acid solution, 2 mL of aqueous plant extract was added. The solutions were then cooled in an ice bath. This was followed by the addition of few drops of sulphuric acid to the solution. The colour change from violet to blue or green indicated the presence of glycosides.

### Test for phlobatannins

To 2 mL of 1% hydrochloric acid solution, 2 mL of aqueous plant extract was added and kept in a water bath at 50 °C. The formation of a red precipitate indicated the presence of phlobatannins.

### Test for saponins

To 5 mL of distilled water, 5 mL of aqueous plant extract was added and warmed. The formation of stable foam indicated the presence of saponins.

### Test for steroids

To 2 mL of aqueous plant extract, 2 mL of chloroform and concentrated acetic acid solution were added along the sides of the test tube.. The formation of a red coloured ring on the upper layer of solution indicated the presence of steroids.

### Test for tannins (ferric chloride test)

To 2 mL of aqueous plant extract, 2 mL of distilled water was added and to this mixture, few drops of ferric chloride solution was added. The formation of a green precipitate indicated the presence of tannins.

### Test for terpenoids (Salkowaski’s test)

To 2 mL of aqueous plant extract, 2 mL of chloroform was added and this was followed by the addition of a few drops of concentrated sulphuric acid. The solution was shaken well. The formation of a yellow coloured lower layer indicated the presence of terpenoids.

### Preparation of poly herbal formulation

The poly herbal formulation contained methanolic leaf extracts of *Nyctanthes arbor-tristis*, unripe fruit pulp of *Aegle marmelos*, the terminal meristem of *Musa paradisiaca* flower, and ripe fruit pulp of *Aegle marmelos* in the ratio 6:2:1:1 (PHF1) and 1:1:1:1 (PHF2). PHF1 was formulated based on the descending order of cumulative DPPH radical scavenging activity and total reducing power of plant extracts.

### Estimation of DPPH radical scavenging activity

DPPH is a purple coloured stable free radical. DPPH assay was performed to estimate the capacity of plant extracts and poly herbal formulations to reduce DPPH to 1,1-diphenyl-2-picryl hydrazine, a colourless compound. Its absorbance was measured at 510 nm [50].

DPPH radical scavenging activity of different plant extracts and poly herbal formulations was determined as described previously [51]. DPPH radical scavenging assay for Poly Herbal Formulation 1 (PHF1) was performed in duplicates. To 2 mL each of methanolic plant extracts, poly herbal formulations and reference standard quercetin at different concentrations were mixed with 3 mL of methanol solution. The reaction mixtures were incubated at room temperature for 15 min in dark and absorbance was measured at 510 nm. DPPH free radical scavenging ability (%) of plant extracts was calculated using the following formula:

$$ \%\mathrm{inhibition}=\left(\left({Absorbance}_{control}-{Absorbance}_{sample}\right)/{Absorbance}_{control}\right)\times 100 $$

### Estimation of total reducing power

Total reducing power assay was performed to estimate the capacity of plant extracts and poly herbal formulations to reduce ferric ion to ferrous ion. The total reducing power of plant extracts and poly herbal formulations was determined using the total reducing power assay. This assay, based on a method previously described [46, 52]*,* estimates the capacity of plant extracts and poly herbal formulations to reduce ferric ion to ferrous ion. Vitamin C (ascorbic acid) was used as a standard. 400 μL of methanolic plant extracts, poly herbal formulations, and ascorbic acid at different concentrations were separately mixed with 1 mL of 0.2 M phosphate buffer (pH 6.6) and 1 mL of 1% potassium ferricyanide. The samples were incubated at 50 °C for 30 min. After the incubation period, 1 mL of 10% trichloroacetic acid was added to the reaction mixtures and centrifuged at 3000 rpm for 10 min. 1 mL of supernatants were then added to 1 mL of distilled water and 170 μL of 0.1% ferric chloride separately, and incubated at 50 °C for 30 min. After the incubation period, absorbance was measured at 700 nm. A standard curve for ascorbic acid was generated and the linear equation was used to calculate the reducing power of the extracts equivalent to vitamin C.

### Determination of anti-elastase activity

Elastase is a protease enzyme that degrades elastin. Inhibition of elastase can prevent skin aging [53]. Anti-elastase assay was performed to determine the ability of the poly herbal formulations to degrade elastase.

Anti-elastase activity of plant extracts and poly herbal formulation 6:2:1:1 (PHF1) were determined spectrophotometrically [54]. The assay was performed in 0.2 M Tris-HCl buffer (pH 8.0). Porcine pancreatic elastase (EC.3.4.21.36) was dissolved in cold 0.2 M Tris-HCl buffer (pH 8.0) to prepare 100 μg/mL of enzyme stock solution, and then diluted to get 10 μg/mL. The substrate, 0.22 mM N-succinyl-Ala-Ala-Ala-p-nitroanilide, was dissolved in 0.2 M Tris-HCl buffer (pH 8.0) to prepare 1 mg/mL substrate solution. Different concentrations of the plant extracts were incubated with the enzyme for 20 min at room temperature before adding the substrate to begin the experiment. The final reaction mixture consisted of 25 μL of various concentrations of plant extracts, 25 μL of substrate, 25 μL of enzyme and 175 μL of Tris-HCl buffer. Copper sulphate solution (100 mM) was used as a positive control and the negative control consisted of Tris-HCl buffer. The absorbance was measured immediately at 405 nm and then continuously for 20 min. Anti-elastase activity of the plant extracts was calculated using the following formula:

$$ \%\mathrm{inhibition}=\left(\left({Absorbance}_{control}-{Absorbance}_{sample}\right)/{Absorbance}_{control}\right)\times 100 $$

### Determination of nitric oxide scavenging activity

Nitric oxide is a gaseous free radical produced by the enzyme nitric oxide synthase during the conversion of arginine to citrulline. Nitric oxide can react with superoxide anion and give rise to powerful free radicals called peroxynitrite radicals [55]. Therefore, inhibiting the ability of nitric oxide to form free radicals can help in the treatment of oxidative skin damage.

Nitric oxide scavenging activity of poly herbal formulations was determined according to a previously described method [56]. The reaction mixture consisted of 200 μL of 10 mM nitroprusside and 200 μL of standard curcumin or poly herbal formulations in phosphate buffer (pH 7.0). This was incubated for 150 min at room temperature. After the incubation period, 500 μL of Griess reagent was added and further incubated for 10 min at room temperature.

Following this, absorbance was measured at 564 nm. The nitric oxide scavenging ability (%) of poly herbal formulations was calculated using the following formula:

$$ \%\mathrm{inhibition}=\left(\left({Absorbance}_{control}-{Absorbance}_{sample}\right)/{Absorbance}_{control}\right)\times 100 $$

### Cell culture

NIH3T3 mouse embryonic fibroblast cells and A375 human malignant melanoma cells were cultured in DMEM media supplemented with 10% fetal bovine serum (FBS) in a humidified atmosphere with 5% CO_2_ at 37 °C. The medium was replaced every two days and the cells obtained from passages between two and four were used in the study.

### In vitro cytotoxicity test

NIH3T3 and A375 cells were seeded in 96 well plates at a density of 1 × 10^4^ cells per well in DMEM (10% FBS) and incubated for 24 h. The cells were then treated with poly herbal formulations of various concentrations in DMEM without FBS from 0 to 320 μg/mL for 24 h. The well containing only DMEM served as a negative control. After incubation, 0.5 mg/mL MTT was added to the wells and incubated for 4 h at 37 °C. The media was removed and formazan crystals were dissolved in 100 μL of DMSO per well, and the absorbance was measured at 570 nm.

### Statistical analysis

Linear regression analysis was performed to calculate the IC_50_ values of standards, plant extracts, and poly herbal formulations. Experimental results were subjected to one-way analysis of variance and linear correlations were analysed using GraphPad Prism 7.0 software with a significance level *P* < 0.05.

## Results

### Preliminary qualitative phytochemical analysis of plant extracts

The methanolic extracts of unripe fruit pulp of *Aegle marmelos*, ripe fruit pulp of *Aegle marmelos*, leaves of *Nyctanthes arbor-tristis,* and the terminal meristem of *Musa paradisiaca* flower were screened for the presence of various bioactive phytochemical compounds. The tests revealed the presence of alkaloids, flavonoids, glycoside, saponins, steroids, and terpenoids in the terminal meristem of *Musa paradisiaca* flower; flavonoids, glycoside, saponins, tannins, and terpenoids in the leaves of *Nyctanthes arbor-tristis*; alkaloids, steroids and flavonoids in unripe fruit pulp of *Aegle marmelos*; steroids and terpenoids in ripe fruit pulp of *Aegle marmelos* (Table 1).

**Table 1:** Results of preliminary phytochemical analysis of methanolic extracts of unripe fruit pulp of *Aegle marmelos*, ripe fruit pulp of *Aegle marmelos*, leaves of *Nyctanthes arbor-tristis,* and the terminal meristem of *Musa paradisiaca* flower. (+: present, −: absent)

Tests performed	Unripe fruit pulp of *Aegle marmelos*	Ripe fruit pulp of *Aegle marmelos*	*Nyctanthes arbor-tristis* leaves	Terminal meristem of *Musa paradisiaca* flower
Test for alkaloids	+	–	–	+
Test for flavonoids	+	–	+	+
Test for glycosides	–	–	+	+
Test for phlobatannins	–	–	–	–
Test for saponins	–	–	+	+
Test for steroids	+	+	–	+
Test for tannins	–	–	+	–
Test for terpenoids	–	+	+	+

### DPPH radical scavenging assay

**Fig. 1:**
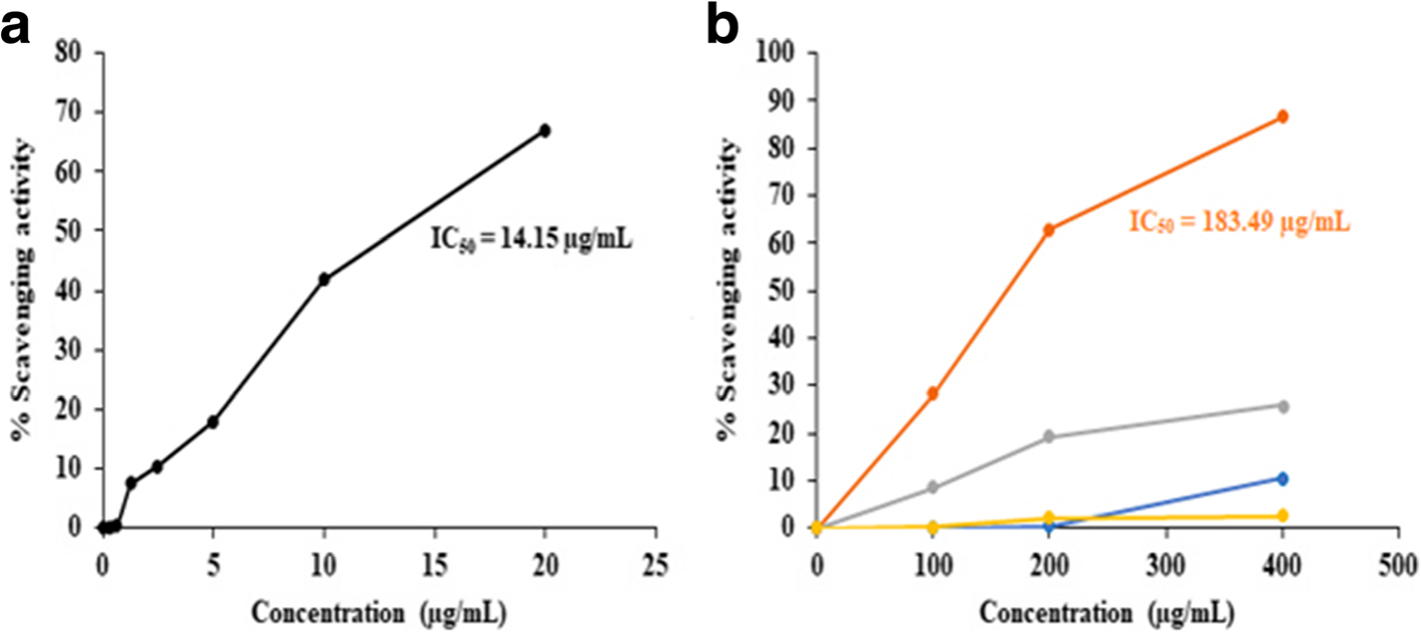
**a** DPPH radical scavenging activity of quercetin. **b** Comparison of DPPH radical scavenging activity of methanolic extracts of *Nyctanthes arbor-tristis* leaves (orange), unripe fruit pulp of *Aegle marmelos* (grey), terminal meristem of *Musa paradisiaca* flower (blue) and ripe fruit pulp of *Aegle marmelos* (yellow)

**Fig. 2:**
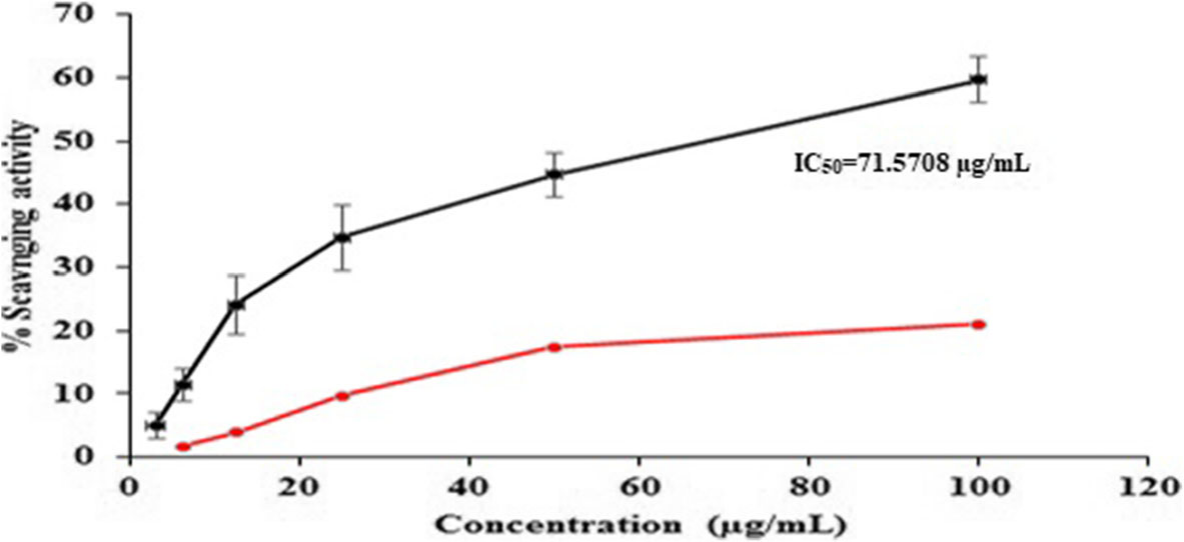
Comparison of DPPH radical scavenging activity of poly herbal formulations. PHF1 (black) and PHF2 (red)

The DPPH radical scavenging activity of methanolic plant extracts and poly herbal formulations was compared with quercetin. The correlation coefficient (*R*^2^) of methanolic extracts of *Nyctanthes arbor-tristis*, unripe fruit pulp of *Aegle marmelos*, ripe fruit pulp of *Aegle marmelos*, terminal meristem of *Musa paradisiaca* flower, PHF1 and PHF2 was found to be 0.9146, 0.9021, 0.7386, 0.9109, 0.9109, and 0.8736, respectively. Among the four plant extracts tested, *Nyctanthes arbor-tristis* exhibited higher DPPH radical scavenging activity (Fig. 1b). The other plant extracts i.e., methanolic extracts of unripe fruit pulp of *Aegle marmelos*, ripe fruit pulp of *Aegle marmelos* and terminal meristem of *Musa paradisiaca* flower exhibited lower DPPH radical scavenging activity (Fig. 1b). The IC_50_ value of *Nyctanthes arbor-tristis* leaves was 183.49 μg/mL and that of quercetin was 14.15 μg/mL (Additional file 1: Table S1). When compared with the individual plant extracts, PHF1 exhibited better DPPH radical scavenging activity with an IC_50_ value of 71.5708 ± 0.2390 μg/mL and PHF1 was also more potent than the PHF2 (Fig. 2) (Additional file 1: Table S2).

### Total reducing power assay

**Fig. 3:**
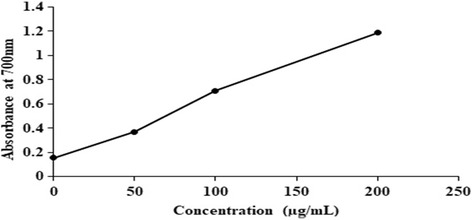
A standard graph of total reducing power of standard vitamin C as a function of concentration (μg/mL) along x- axis and absorbance at 700 nm along y-axis

**Fig. 4:**
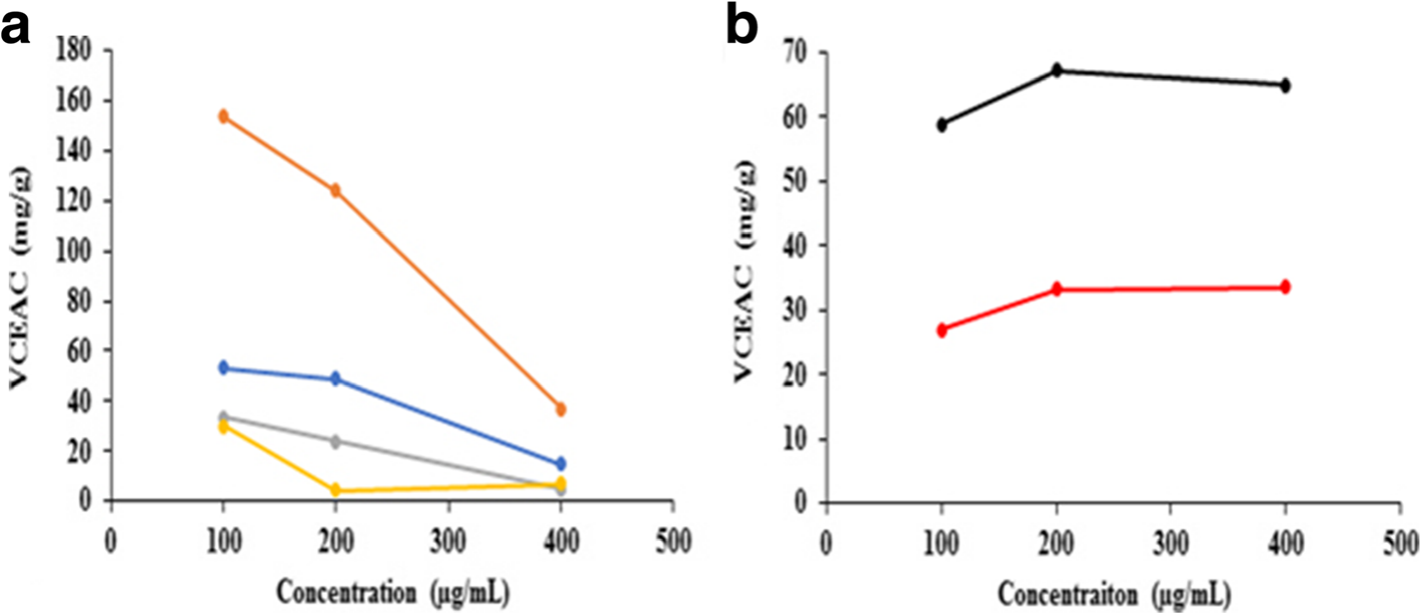
**a** Comparison of total reducing power of methanolic extracts of *Nyctanthes arbor-tristis* leaves (orange), unripe fruit pulp of *Aegle marmelos* (grey), terminal meristem of *Musa paradisiaca* flower (blue) and ripe fruit pulp of *Aegle marmelos* (yellow) in terms of VCEAC at various concentrations. **b** Comparison of total reducing power of methanolic extracts of poly herbal formulations: PHF1 (black) and PHF2 (red) in terms of VCEAC at various concentrations

**Fig. 5:**
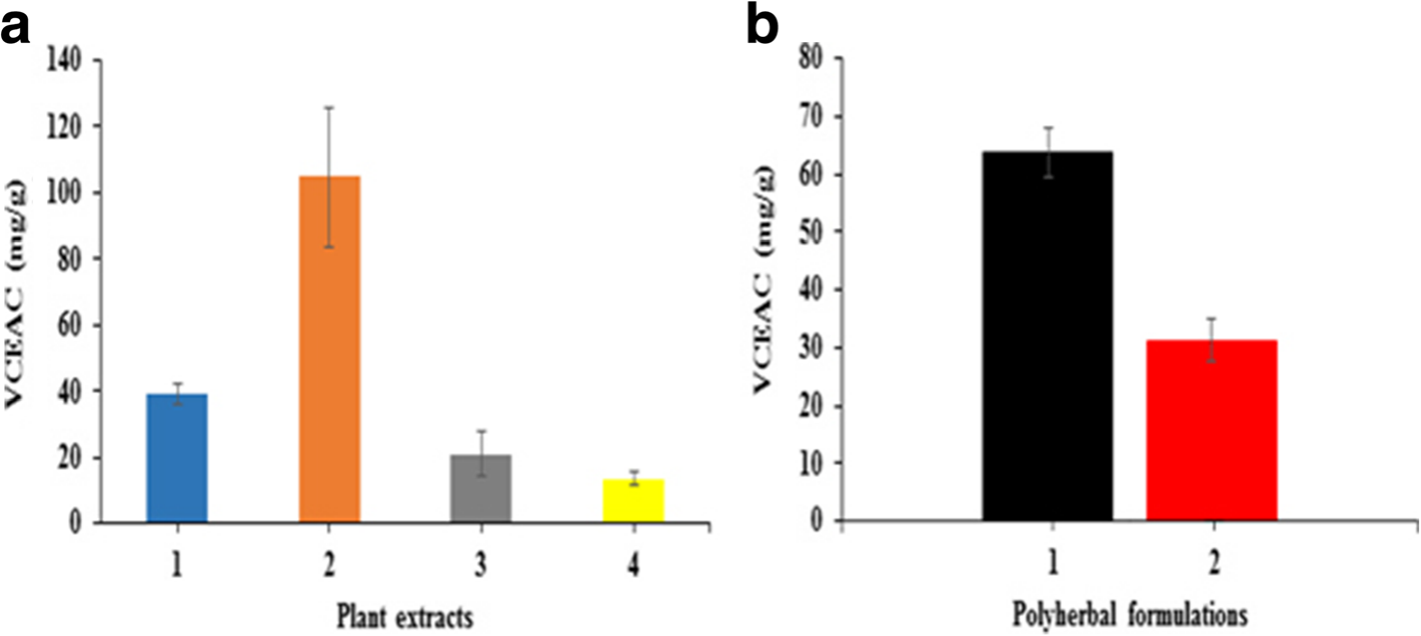
**a** Comparison of total reducing power of methanolic extracts of terminal meristem of *Musa paradisiaca* flower (1), *Nyctanthes arbor-tristis* leaves (2), unripe fruit pulp of *Aegle marmelos* (3), and ripe fruit pulp of *Aegle marmelos* (4) in terms of VCEAC. **b** Comparison of total reducing power of methanolic extracts of poly herbal formulations PHF1 (1) and PHF2 (2) in terms of VCEAC

The total reducing power of methanolic plant extracts and poly herbal formulations were compared with standard vitamin C. A standard graph of absorbance at 700 nm versus concentration of vitamin C was obtained from which vitamin C equivalent anti-oxidant capacity of various plant extracts and poly herbal formulations were calculated (Fig. 3). A dose- dependent reducing power was observed for methanolic plant extracts and poly herbal formulations (Fig. 4). The correlation coefficient (*R*^2^) of methanolic extracts of *Nyctanthes arbor-tristis*, unripe fruit pulp of *Aegle marmelos* and terminal meristem of *Musa paradisiaca* flower was found to be 0.9503, 1 and 0.9499, respectively. Among the four methanolic extracts tested, Nyctanthes *arbor-tristis* leaves exhibited strongest reducing power of 104.83 ± 21.04 vitamin C mg/100 g of Vitamin C equivalent anti-oxidant capacity (VCEAC) than the other three extracts. The other three extracts exhibited only minimal reducing power (Fig. 5) (Additional file 1: Table S3). The poly herbal formulation PHF1 exhibited higher reducing power of 63.67 ± 4.28 vitamin C mg/100 g of VCEAC, while PHF2 exhibited lower reducing power (Fig. 5) (Additional file 1: Table S4).

### Anti-elastase assay

**Fig. 6:**
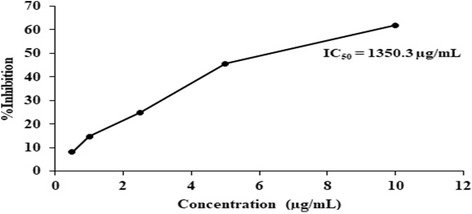
A standard graph of elastase inhibition capacity (y-axis) of standard copper sulfate as a function of concentration (μg/mL) (x- axis)

**Fig. 7:**
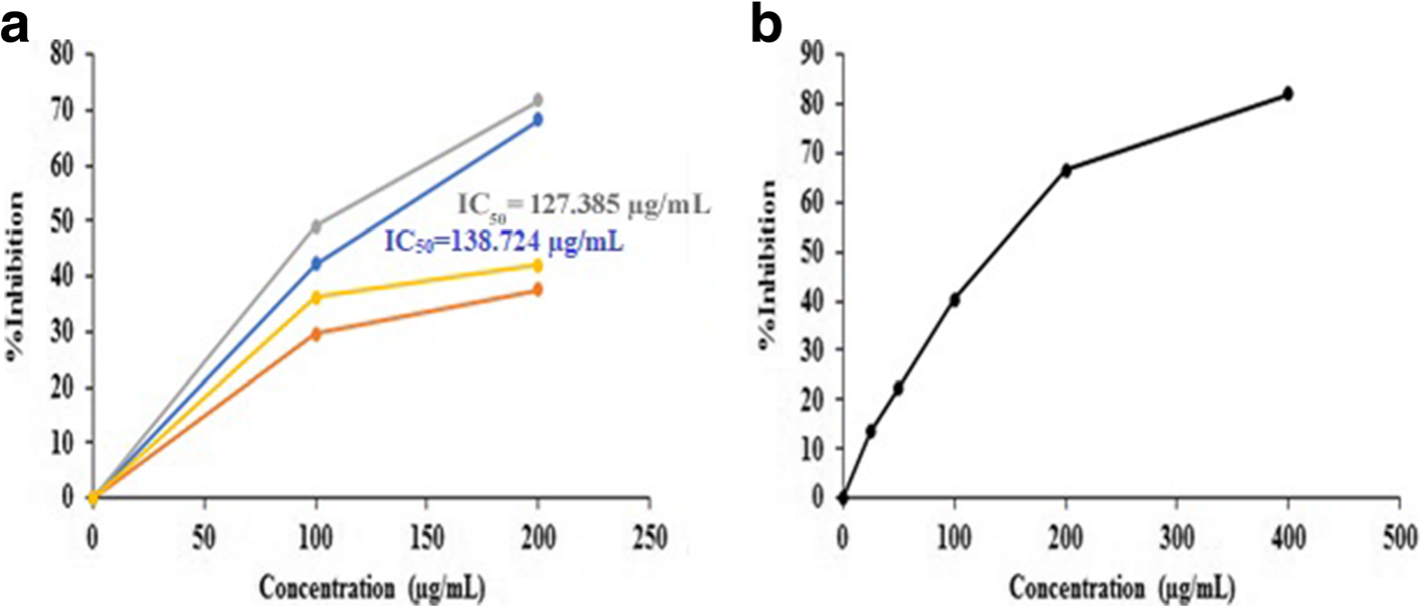
**a** Comparison of elastase inhibition capacity of methanolic extracts of *Nyctanthes arbor-tristis* leaves (orange), unripe fruit pulp of *Aegle marmelos* (grey), terminal meristem of *Musa paradisiaca* flower (blue) and ripe fruit pulp of *Aegle marmelos* (yellow). **b** Elastase inhibition capacity of PHF1 (y-axis) at various concentrations (x-axis)

The elastase inhibition capacity of methanolic plant extracts and PHF1 was compared with standard copper sulfate. From the previously mentioned anti-oxidant assays, it was observed that PHF2 possessed lower anti-oxidant capacity. Hence, its elastase inhibition capacity was not tested. A standard graph of elastase inhibition capacity of copper as a function of concentration was obtained (Fig. 6). Among the four plant extracts tested, unripe fruit pulp of *Aegle marmelos* and terminal meristem of *Musa paradisiaca* flower exhibited good elastase inhibition capacity with IC_50_ values 127.385 μg/mL and 138.724 μg/mL, respectively. The other two plant extracts exhibited lower elastase inhibition capacity. Although the unripe fruit pulp of *Aegle marmelos* was the most potent with an IC_50_ value of 127.385 μg/mL, PHF1 with an IC_50_ value of 172.1 μg/mL is still a promising agent as it is more potent than the standard, copper sulfate (Fig. 7) (Additional file 1: Table S5).

### Nitric oxide scavenging assay

**Fig. 8:**
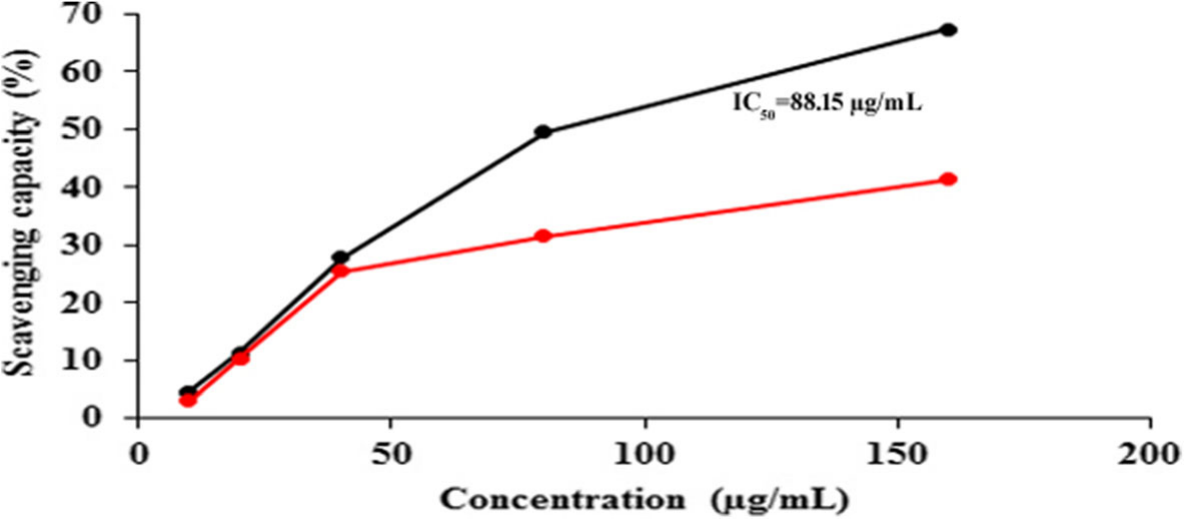
Nitric oxide scavenging capacity of poly herbal formulations: PHF1 (black) and PHF2 (red)

The nitric oxide scavenging capacity of poly herbal formulations was compared with curcumin. PHF1 exhibited higher nitric oxide scavenging capacity than PHF2, but was lower than of curcumin, and the IC_50_ value of PHF1 was 88.15 μg/mL (Fig. 8) (Additional file 1: Table S6).

### MTT assay

**Fig. 9:**
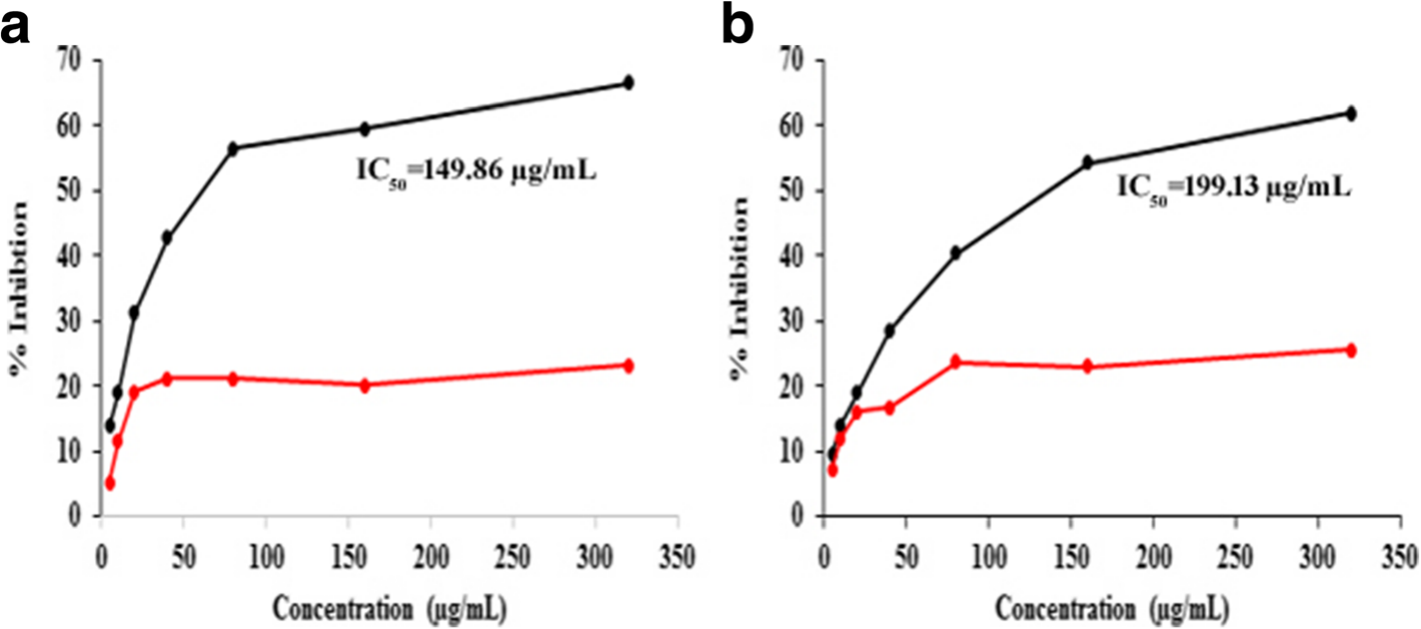
**a** Inhibitory capacity of PHF1 (black) and PHF2 (red) when tested against NIH3T3 cell line. **b** Inhibitory capacity of PHF1 (black) and PHF2 (red) when tested against A375 cell line

Poly herbal formulations were tested for its inhibitory capacity against normal fibroblast cell line NIH3T3 and human malignant melanoma cell line A375. Both the formulations (PHF1 and PHF2) exhibited dose-dependent inhibitory activity in the two tested cell lines, although PHF2 was much less potent than PHF1. When tested against NIH3T3 cells, the highest amount of inhibition was seen for PHF1 (66.57%), with an IC_50_ value of 149.86 μg/mL, whereas PHF2 exhibited lower inhibitory activity (23.25%) (Table 8). Moreover, PHF1 was more potent than PHF2 (Fig. 9a). When tested against human malignant melanoma A375 cell line, PHF1 exhibited higher inhibitory activity (61.88%) with an IC_50_ value of 199.13 μg/mL, than PHF2 (Fig. 9b) (Additional file 1: Table S7).

## Discussion

Normal cellular metabolism and influence of internal and external factors on cellular metabolism leads to the formation of ROS in skin [8, 57, 58]. Some of the sources of ROS in epidermal and dermal layers of skin are cytochrome oxidase, iron ions, electron transport chain, xanthine oxidases, peroxisomal oxidases, lipoxygenases, cytochrome P450, cyclo-oxygenase, and NADPH oxidase [57]. These non-enzymatic and enzymatic sources of ROS, responsible for the production of superoxide anions, peroxyl radicals and hydroxyl radicals [57], can oxidize nucleic acids, proteins, and lipids to cause skin damage [8]. Anti-oxidants reverse the damages caused by ROS by neutralizing ROS. However, antioxidants are present in lower concentrations in cells and therefore must be obtained from food. Some of the anti-oxidants found in epidermal and dermal layer of skin are Co-enzyme Q10, catalase, glutathione peroxidase, and superoxide dismutase [57]. Non-enzyme anti-oxidants such as vitamin C and vitamin E can be obtained from food. Other anti-oxidants such as glutathione, uric acid, beta carotene, ascorbate, and small proline molecules are produced in low concentrations in the body and are not sufficient to neutralize the free radicals.

A number of phenolic compounds are present in plants such as simple phenolics, phenolic acids, anthocyanins, and flavonoids [59]. The anti-oxidant activity of phytochemicals such as phenolic acids and flavonoid is elicited via free radical scavenging and increased levels of anti-oxidant enzymes in plasma [60]. Since the above-mentioned compounds and also other phytochemicals possess the ability to act as free radical scavengers [59] and more importantly, present in dietary sources, the investigation of the anti-oxidant activity of these compounds against free radical-induced skin damage holds great promise in the development of safe, efficacious, and less toxic drugs for anti-aging skin therapy.

In this study, the anti-oxidant activity of *Nyctanthes arbor-tristis* leaves, unripe and ripe *Aegle marmelos* fruit pulp, and terminal meristem of *Musa paradisiaca* flower was investigated. Numerous important phytochemicals such as flavonoids, alkaloids and glycosides were present in the methanolic extract. The anti-oxidant and free radical scavenging activity of the three plants tested are in accordance with results from previous studies using plants of the same genus [61–65], although the methanolic extract from only one of the plants tested exhibited the most potent activity. The results obtained from DPPH radical scavenging assay and nitric oxide scavenging assay confirmed the highest free radical scavenging capacity of *Nyctanthes arbor-tristis*. Unripe and ripe *Aegle marmelos* fruit pulp and terminal meristem of *Musa paradisiaca* flower exhibited less anti-oxidative capacity than *Nyctanthes arbor-tristis*. Based on the anti-oxidant capacities of each plant, PHF1 and PHF2 were formulated and their anti-oxidative capacity was tested using equimolar concentrations. The results obtained from DPPH radical scavenging assay, nitric oxide scavenging assay, reducing power assay and anti-elastase assay confirmed the highest free radical scavenging capacity and elastase inhibitory capacity of PHF1. Studies using other plant extracts have reported that the reduction of ferric ion to ferrous ion acted as an indicator of the presence of reductants. These reductants, via their anti-oxidative property, terminate the free radical chain reaction and aid in the formation of a more stable product by donating electrons to free radicals [66, 67]. In accordance with above-mentioned results, PHF1 exhibited potent reducing power property. It has also been reported that anti-oxidants present in plant extracts are nitric oxide scavengers and act as a competitive inhibitor of oxygen, leading to reduced production of nitrite and nitrate [68, 69]. Since PHF1 exhibited good nitric oxide scavenging activity, its mechanism of action could also be attributed to reduced production of nitrates and nitrites by competing with oxygen. Since elastase causes wrinkles and aging in skin by degrading elastin, an assessment of anti-elastase activity of a plant extract can be a useful indicator of its potential application in cosmetic agents. PHF1 exhibited potent anti-elastase activity when compared to PHF2 and this anti-elastase activity of PHF1 could be attributed to the presence of tannins and flavonoids, as reported in previous studies wherein flavonoid compounds and tannins exhibited significant elastase inhibitory property [70, 71]. Though PHF1 exhibited dose-dependent toxicity (66.57% inhibition) against fibroblasts cells at the highest dose (320 μg/mL) tested, it still could be used as a topical agent in skin anti-aging creams using lower doses to reduce toxicity. Since PHF1 was also cytotoxic against malignant melanoma cells, it has the potential to be developed as an anti-cancer agent with further studies. Though PHF2 was less toxic to fibroblast cells, it exhibited low anti-oxidant capacity, anti-elastase capacity and was also less cytotoxic to malignant melanoma cells. Hence, it does not have any potential for further investigations.

The current study has some limitations. The different assays such as DPPH radical scavenging assay, total reducing power, anti-elastase activity, and nitric oxide scavenging activity using the plant extracts and PHFs were performed and the activities determined only via biochemical assays. A more accurate representation of the results would be to evaluate the results obtained further in cell culture models by inducing oxidative stress, as reported in a study by Ben Mansour et al. [72]. Moreover, results obtained from the biochemical assays may not always correlate with those obtained using cellular assays as biochemical assays do not replicate the environmental milieu of a cell. Although the MTT assay using PHF1 demonstrated that it had cytotoxic effect against melanoma cancer cell line, it is difficult to prove that it possesses anti-cancer activity without performing further mechanistic studies. A time- and dose-dependent study using cancer cell lines is warranted to investigate how PHF1 elicits cytotoxicity to cells. Furthermore, a preliminary toxicity study in in vivo models should be performed before testing its effect in animal models, if favorable results are obtained from cell culture studies. Another drawback of the study is that only methanolic extract was used throughout the study to test the efficacy of the plant extract and PHF. Ugochukwu et al., reported in their study that the phytochemicals present in the plant extract were present in different concentrations in different solvents [47]. Hence, it is possible that the phytochemicals present in the three plants tested may have been obtained in more potent forms had different solvents been used during the extraction process.

In future studies, the individual phytochemicals from the plant extracts could be extracted, characterized and further studies could be performed using in vivo disease models that have lower enzymatic activity of anti-oxidant enzymes such as super oxide dismutase and catalase [73]. Moreover, the anti-cancer activity of PHF1 should be investigated in detail. Further in vitro studies are warranted to study the mechanism of action of the PHF1 at lower doses and then perform in vivo studies to investigate its efficacy and toxicity.

## Conclusion

In conclusion, the anti-oxidant capacity of phytochemicals present in the leaves of *Nyctanthes arbor-tristis* and PHF1 can be used as a potential agent to prevent skin aging and restore skin elasticity. Moreover, PHF1 holds promise as a potential anti-cancer agent in the treatment of malignant melanoma. Further studies are warranted to purify and characterize the phytochemicals present in plant extracts and investigate the anti-cancer activity of PHF1 in detail using cell culture models.

## Additional file

Additional file 1: **Table S1.** DPPH radical scavenging activity of plants extracts and quercetin. The supplementary file compares the DPPH radical scavenging activity of different methanolic plant extracts in terms of its percentage of inhibition and IC_50_ values. Quercetin was used as a standard to compare the efficacy of the plant extracts. **Table S2**. DPPH radical scavenging activity of poly herbal formulations. The supplementary file compares the DPPH radical scavenging activity of poly herbal formulations in terms of its percentage of inhibition and IC_50_ values. **TableS3**. Total reducing power of methanolic plant extracts. The supplementary file compares the ability of plant extracts to reduce ferric iron to ferrous iron, using vitamin C as a standard to calculate the reducing power of the extracts equivalent to vitamin C. **Table S4**. reducing power of poly herbal formulations. The supplementary file compares the ability of poly herbal formulations to reduce ferric iron to ferrous iron, using vitamin C as a standard to calculate the reducing power of the extracts equivalent to vitamin C. **Table S5**. Elastase inhibition capacity. The supplementary file compares the elastase inhibition capacity of plant extracts and PHF1 with copper sulfate as standard. **Table S6**. Nitric oxide scavenging capacity. The supplementary file compares the nitric oxide scavenging capacity of plant extracts and poly herbal formulations with curcumin as standard. **Table S7**. In vitro inhibitory capacity of poly herbal formulations. The supplementary file enlists the percentage of cells inhibited by poly herbal formulations at different concentrations against NIH3T3 fibroblast cells and A375 malignant melanoma cells (PDF 727 kb)

## Abbreviations

DMEM: Dulbecco’s Modified Eagle Medium
DMSO: Dimethyl Sulfoxide
DPPH: 2, 2-Diphenyl-1-picrylhydrazyl
FBS: Fetal Bovine Serum
MTT: 3-(4, 5-Dimethyl-2-thiazolyl)-2,5-diphenyl-2H-tetrazolium bromide
PHF1: Poly Herbal Formulation 1
PHF2: Poly Herbal Formulation 2
R^2^: correlation coefficient
ROS: Reactive Oxygen Species
VCEAC: Vitamin C Equivalent Anti-oxidant Capacity
