# Supplementary material for: Poly herbal formulation with anti-elastase and anti-oxidant properties for skin anti-aging
Source: BMC Complement Altern Med. 2018 Jan 29;18:33. doi: 10.1186/s12906-018-2097-9 (PMC5789588; doi:10.1186/s12906-018-2097-9)
Supplement: Additional file 1: — Table S1. DPPH radical scavenging activity of plants extracts and quercetin. The supplementary file compares the DPPH radical scavenging activity of different methanolic plant extracts in terms of its percentage of inhibition and IC50 values. Quercetin was used as a standard to compare the efficacy of the plant extracts. Table S2. DPPH radical scavenging activity of poly herbal formulations. The supplementary file compares the DPPH radical scavenging activity of poly herbal formulations in terms of its percentage of inhibition and IC50 values. TableS3. Total reducing power of methanolic plant extracts. The supplementary file compares the ability of plant extracts to reduce ferric iron to ferrous iron, using vitamin C as a standard to calculate the reducing power of the extracts equivalent to vitamin C. Table S4. reducing power of poly herbal formulations. The supplementary file compares the ability of poly herbal formulations to reduce ferric iron to ferrous iron, using vitamin C as a standard to calculate the reducing power of the extracts equivalent to vitamin C. Table S5. Elastase inhibition capacity. The supplementary file compares the elastase inhibition capacity of plant extracts and PHF1 with copper sulfate as standard. Table S6. Nitric oxide scavenging capacity. The supplementary file compares the nitric oxide scavenging capacity of plant extracts and poly herbal formulations with curcumin as standard. Table S7. In vitro inhibitory capacity of poly herbal formulations. The supplementary file enlists the percentage of cells inhibited by poly herbal formulations at different concentrations against NIH3T3 fibroblast cells and A375 malignant melanoma cells (PDF 727 kb) [file 12906_2018_2097_MOESM1_ESM.pdf]

Supplementary Table 1 DPPH radical scavenging activity of plants extracts and quercetin

Methanolic plant extracts	Concentration (µg/mL)	% Inhibition	IC_{50} value (µg/mL)
Quercetin (Standard)	0.31	0.00	14.15
	0.63	0.36	
	1.25	7.49	
	2.50	10.42	
	5.00	18.07	
	10.00	41.83	
	20.00	67.07	
Nyctanthes arbor-tristis leaves	100	28.19	183.49
	200	62.75	
	400	86.51	
Unripe fruit pulp of *Aegle marmelos*	100	8.57	50% inhibition not achieved
	200	19.18	
	400	25.92	
Ripe fruit pulp of *Aegle marmelos*	100	0.14	
	200	2.05	

	400	2.49	50% inhibition not achieved
Terminal meristem of *Musa paradisiaca* flower	100	0	50% inhibition not achieved
	200	0.36	
	400	10.42	

Supplementary Table 2 DPPH radical scavenging activity of polyherbal formulations

**Polyherbal formulations**	**Concentration (µg/mL)**	**% Inhibition**	**IC_{50} value (µg/mL)**
PHF1	3.125	4.9671 ± 2.0748	71.5708
	6.25	11.4135 ± 2.6212	
	12.5	24.0851 ± 4.7307	
	25	34.6532 ± 5.2796	
	50	44.6458 ± 3.4871	
	100	59.6359 ± 3.6262	
PHF2	6.25	1.7	50% inhibition not achieved
	12.5	3.8	
	25	9.7	
	50	17.4	
	100	21	

Supplementary Table 3 Total reducing power of methanolic plant extracts

**Methanolic plant extracts**	**Concentration (µg/mL)**	**VCEAC (mg/g)**	**Total reducing power/ VCEAC (vitamin C mg/100 g)**
Nyctanthes arbor-tristis leaves	100	135.50	104.83 ± 21.04
	200	123.75	
	400	37.25	
Unripe fruit pulp of *Aegle marmelos*	100	33.75	20.88 ± 6.81
	200	24.13	
	400	4.75	
Ripe fruit pulp of *Aegle marmelos*	100	30.00	13.67 ± 1.94
	200	4.12	
	400	6.88	
Terminal meristem of *Musa paradisiaca* flower	100	53.25	39.00 ± 3.18
	200	48.75	
	400	15	

Supplementary Table 4 Total reducing power of polyherbal formulations

**Polyherbal formulations**	**Concentration (µg/mL)**	**VCEAC (mg/g)**	**Total reducing power/VCEAC (vitamin C mg/100 g)**
PHF1	100	58.88	63.67 ± 4.28
	200	67.13	
	400	65	
PHF2	100	27	31.25 ± 3.68

Supplementary Table 5 Elastase inhibition capacity of copper sulfate, methanolic plant extracts, and PHF1 with PHF1 having correlation coefficient (R^2) of 0.8997

**Methanolic plant extracts**	**Concentration (µg/mL)**	**Elastase inhibition capacity (%)**	**IC_{50} value**
Copper sulfate (Standard)	0.5	8.16	1350.3 µg/mL
	1.0	14.71	
	2.5	24.88	
	5.0	45.69	
	10.0	61.98	
Nyctanthes arbor-tristis leaves	100	29.71	Less active
	200	37.70	
Unripe fruit pulp of *Aegle marmelos*	100	49.03	127.385 µg/mL
	200	71.68	
Ripe fruit pulp of *Aegle marmelos*	100	36.33	Less active
	200	42.02	
Terminal meristem of *Musa paradisiaca* flower	100	42.23	138.724 µg/mL
	200	68.29	
	25	13.59	

PHF1	50	22.43	172.1 μg/mL
	100	40.29	
	200	66.53	
	400	81.96	

Supplementary Table 6 Nitric oxide scavenging capacity of curcumin and polyherbal formulations with PHF1 and PHF2 having correlation coefficients (R^2) of 0.9283 and 0.8336

**Methanolic plant extracts**	**Concentration (µg/mL)**	**Scavenging capacity (%)**	**IC_{50} values (µg/mL)**
Curcumin	6.25	5.34	30.38
	12.5	12.39	
	25	28.33	
	50	49.92	
	100	53.53	
	200	68.64	
PHF1	10.0	4.5	88.15
	20.0	11.4	
	40.0	27.8	
	80.0	49.5	
	160.0	67.4	
PHF2	10.0	3.1	
	20.0	10.4	
	40.0	25.4	

	80.0	31.5	50% inhibition not achieved
	160.0	41.4	

Supplementary Table 7 Inhibitory capacity of polyherbal formulations against NIH3T3 (t-test: $P < 0.05$) and A375 cell lines

**Cell line**	**Polyherbal formulations**	**Concentration (µg/mL)**	**% Inhibition**
NIH3T3	PHF1	5	13.92
		10	19.06
		20	31.26
		40	42.83
		80	56.53
		160	59.53
		320	66.57
	PHF2	5	5.27
		10	11.50
		20	19.22
		40	21.16
		80	21.23
		160	20.26
		320	23.25

A375	PHF1	5	9.64
		10	14.13
		20	19.06
		40	28.69
		80	40.47
		160	54.24
		320	61.88
	PHF2	5	7.36
		10	11.96
		20	16.14
		40	16.77
		80	23.67
		160	23.02
		320	25.55

Office: 080-2656[illegible]
Fax: 080-2657[illegible]
Email: nadri.bengaluru1@gmail.com

रीजनल आयुर्वेद रिसर्च इन्स्टिट्युट फॉर मेटाबॉलिक डिसॉर्डर्स
(केन्द्रीय आयुर्वेदीय विज्ञान अनुसंधान परिषद, आयुष मंत्रालय, भारत सरकार, नई दिल्ली)
सरकारी केन्द्रीय औषधालय, अशोक स्तंभ, जयनगर बेंगलूर- ५६००११

REGIONAL AYURVEDA RESEARCH INSTITUTE FOR METABOLIC DISORDERS
(Central Council for Research in Ayurvedic Sciences, Ministry of AYUSH, Govt. of India, New Delhi)
G.C.P.Annexe, Ashoka Pillar, Jayanagar, Bangalore-560 011

प्रमाणीकरण/औ.पौ.स.ई./आर.ए.आर.ऐ.एम.डि./बेंगलुरू/2016-17/121 दिनांक /Date: 04.05.2016
Authentication / S.M.P.U. / R.A.R.I.M.D. / BNG/2016-17/121

प्रमाणपत्र / Certificate

दीपिका डि सीरंगि एवं इंदुजा के, स्नातक के छात्र, बयोटेकनालोजि विभाग, दयानंद सागर कालेज, बेंगलुरु के द्वारा प्रस्तुत किया गया पौधे का नमूने को नीचे लिखा हुए नाम से पहचान के प्रमाणीकृत किया जाता है।

This is to certify that the plant samples were submitted by **Miss. Deepika D Sarangi and Miss. Induja K**. Under Graduate students of 8th semester B E Biotechnology, Dayananda Sagar College of Engineering, Bangalore have been identified and authenticated as below:

1. *Musa paradisiaca* L. Family: Musaceae **(RRCBI- Mus. 144)**
2. *Aegle marmelos* (L.) Correa Family: Rutaceae **(RRCBI- 12951)**
3. *Nyctanthes arbor-tristis* L. Family: Oleaceae **(RRCBI- 14086)**

द्वारा पहचाना /Identified by
(डॉ. सिद्दमल्लय्या/Dr. Shiddamallayya N)

द्वारा प्रमाणीकृत /Authenticated by
(डॉ. वि. रामा राव/Dr. V. Rama Rao)

अ.अ.(वैज्ञानिक-3) प्रभारी/R.O. (Scientist-3) i/c
(Dr. Sulochana Bhat)

को/To,
Dr. Sahabudeen S,
Associate professor,
Department of Biotechnology,
Dayananda Sagar College of Engineering,
Bangalore
